# Miasmatic Paralytic Fever, or Pernicious Malarial Fever

**Published:** 1895-06

**Authors:** D. L. Peeples

**Affiliations:** Navasota


### TEXAS MEDICAL JOURNAL.

ESTABLISHED JULY, 1885.

PUBLISHED MONTHLY.—SUBSCRIPTION $2.00 A YEAR.

VOL. X. AUSTIN, JUNE, 1895. NO. 12.

### Original Contributions.

For Texas Medical Journal.

### MIASMATIC PARALYTIC FEVER, OR PERNICIOUS MALARIAL FEVER.

BY D. L. PEEPLES, M. D., NAVASOTA.

RECOGNIZING the minimum amount of literature on this all important and most formidable departure amongst the forms of malarial fevers, I can but feel constrained to attempt a small contribution of experience, or personal observation concerning this dreaded disease. In typical cases, the first paroxysm jeopardizes life to such an extent, that it not unfrequently goes beyond the controlling influence of all appropriate remedial agents. The secondary is but the relentless clasping mortuary shackles.

The tertiary causes man to stand in a state of awe, confused and dependently gazing upon the mysteries of God, developing a metamorphosis, subsequently and without loss of time, rendering carnality to assume its spiritual and eternal form. I have already commented, in regard to the dearth of literature on this subject, as far as my knowledge goes, and necessarily my observation forces me to assume a position differing materially from the writings of our authors in the text-books, concerning certain descriptive characteristic features of this disease, probably due to the locations of the different malarial districts. However, to describe this disease as we see it here, I, as I have already stated, must deviate in my semeiography, and also indulge in some hypothetical pathology, as it was not yet clear. It is to be regretted, that our best authors have never had a case of miasmatic paralytic fever to fall under their observation. The condition alluded to will subsequently be taken under consideration in my nomenclature. If I am not assuming a position of pedantry or ostentation, I would in my inaccurate and practical way, suggest for consideration an appropriate epithet for this disease prevailing in our Southern clime. The appellation, pernicious, could scarcely be more appropriate or expressive of its gravity, but the epithet I desire to present is quite descriptive of the disease itself; e. i. "Miasmatic paralytic fever," as designated previously.

My ascription of the adjective, paralytic, will be explained all along in its application. The internal viscera, the nervous and circulatory systems rapidly succumb to a state of syncope from a peculiar influence of malarial virulence, if not promptly and efficiently met. This phenomenal and powerful morbid influence immediately begins to preside over the nervous system, resulting in muscular peristaltic paresis.

Strange, but true, both extremities of the alimentary canal retain their functional muscular activity, while a profound paralytic state exists below the pylorus, and above the ileo-cæcal valve. A constant hypertonic muscular contractility of the stomach results from a reflex irritability, also biliary distention, local and foreign ecchymosis. The alimentary canal is almost entirely coated with a thick viscid and elastic mucus, of different hues and characters, rendering the absorptive function very defective indeed, and at times completely lost. We must have visceral paralysis before confirming our diagnosis of typical cases of miasmatic paralytic fever. It may be congruous to state that I have a most prolific field for malarial study. There is no occlusion, obstruction, adhesion, strangulation, loops, intussusception, fold, hernia, nor any spasm, present to develop this phenomenal inertia. Therefore visceral paralysis positively exists, as the evidence is abundant, and space will not permit further discussion demonstrating this fact, I shall now indulge in its brief hypothetical pathogenetic etiology. It is a condition, a fixed state of the nervous system, originating from the toxiferous effects of malaria. It is highly probable that the floor of the fourth ventricle is in a state of hyperæmia, or covered with minute extravasations, and also an ecchymotic state of other sections of the medulla oblongata tending to produce antiperistatis of the stomach, and developing this phenomenal suspension of peristaltic function. It is not to be forgotten that all the spinal gangliæ eventually reach this pathological state with the exception of the two lower pair, and probably the impar ganglion. Recognizing the centrifugal generation of impulses through the right par-vagum and taking for granted, the experiments of Brachet resulted in any conclusive facts, then the confirmation of this pathological hypothesis is still more evident. At this stage the heart is thrown into a most terribly cacosfixiated condition, condition enormously increasing the frequency of its pulsations, diminishing its volume and force through efferent impulses from central irritation at the pneumogastric origin. The respiratory organs become characteristically impeded, labored, nervous, and here the Cheyne Stokes' respiration is vividly pictured. The lungs are in a state of hypostatic congestion, rapidly filling with a viscid tenacious mucous, which soon results in an asphyxiated dyspneaic condition as the end approaches, termed "the death rattle." The non-medullated nerve-fibers and filaments, or the plexuses of Auerbach and Meissner terminate in paresis, therefore, this vermiculation is lost. The experiments of Muller will render this morbid condition pathognomonic, or sufficient to demonstrate the facts I have attempted to prove. The revelation in the discovery of the vaso-motor nerves, by Brown-Sequard, is of considerable interest to the pathologist when he studies the morbid influence of malaria over the sympathetic nervous system governing calorification, nutrition and secretion. The blood of an infected patient who resides in a fertile malarial district is a field of mnltiplication for the exceedingly rich hæmatozoons of Laveran, or the plasmodium, which proves to be a rapid and violent poison to the functions of innervation. To recapitulate slightly, would say from my observations, that there is an accumulation of hæmatozoonous blood, if I may so speak, which rapidly results in capillary statis around the various spinal and other gangliæ. All the filaments, or ganglionic roots of the great sympathetic, become in a state of ecchymosis charged, with teeming millions of plasmodium in their destructive richness, and but little invigorating, or oxygenized blood to restore this functional depreciating innervation, which facilitates the development of this phenomenal paralytic peristalsis. This paresis and the thick viscid mucus contained in the alimentary avenue, will establish to some extent the specious idea of the non-physiological influence of the most powerful purgatives along the course of the ten million villi of the canal.

The liver and spleen are congested to some incomprehensible extent, but owing to their extreme softness and vascularity, especially the latter, it is indeterminate on palpation, as there is no induration defining the borders.

Etiology.—This disease originates strictly from malaria, being non-contagious, non-transmissible from one person to another, except by inoculation, and attacking either sex, all ages, and races, the negro being less prone to succumb, on exposure. The elucidation of the properties and habitat of malaria at the present day, by scientists, is not yet satisfactory, but suffice it to say, it is one of the lower forms of life, probably of the amœboid type. The amœbæ have been discovered in drops of blood taken from a malarial patient. The divisibility of malaria into the bacillus malariæ of Klebs and the micrococci, etc., will not be discussed for want of space. The principal point in favor of these existing living amœbic organism is their analogous amœboid movements, to that of the white blood-corpuscles, only possessing greater activity. It is highly probable that the day is not far distant when these malarial molecular organisms will be considered more of a parasitic or amœbic character than bacteriologic. However and whatever malaria may be, it is introduced into the system through the respiratory organs, in fruits, unsterilized surface water, and speciously there is to some extent, an osmotic process of infection through the porocity of the epidermis.

### SYMPTOMATOLOGY.

The symptomatic course of miasmatic paralytic fever is here detailed, but not by diagnostic discrimination, though, if space permitted, a beautiful opportunity would be happily utilized in the classification of malarial fevers, designating this as a distinct type, arising from the same cause, and intensified by climatic influences. Occasionally a premonitory choleraic state is observed, but as the disease advances, synchronously the bowels are paralyzed, hence a cessation of peristalsis, but a continuation of nausea and vomiting.

The onset may or may not be ushered in with a chill, but its peculiarity is characteristically a phenomenon. A full prolonged and quivering inspiration is comparatively frequent, and carries with it a slight nervous hissing vibration. Chilliness at times and flashes of heat are realized, with pallor and lividity of the face. The skin generally cyanotic, the surface cold, clammy and especially the extremities. The trunk is sometimes hot, the extremities vice versa. The eyes are bright and clear, with dark and ecchymotic rings below them. The expression depicts alarm, looks pinched, worn, fatigued, and deathly, though the mind almost invariably remains perfectly rational until death. Visceral paresis is thoroughly developed, even beyond the influences of the most powerful purgations, and mechanical means that may be utilized. The heart becomes disturbed and rapidly yielding to the toxic influence of miasm. The kidneys are generally undisturbed, though scantiness, and rarely retention, may occasionally be observed. The temperature generally ranges from 99° to 102° F., sometimes higher, and if the exacerbation is not too excessive, is a favorable indication. Patietns lie in the dorso-decubitus position. As the disease progresses he turns on his side, not by preference, but for convenience and protection to avoid asphyxia in his vomit. Not infrequently the tongue is unaltered as to its previous state, and especially so in the early stages, though sometimes dry, pale and cold. Sometimes resembles a sliced blood beet, again brown and heavily coated, occasionally black. Generally there is oppression to a great degree of uneasiness over the epigastric region, but no pain; probably due to weakness and approaching respiratory failure. A burning heat is not unfrequently appreciated in the stomach, with excessive and almost insatiable thirst. The patient is heard with incessant cries for ice, ice water, etc. He feels and exclaims in pitiable tones his inability to imbibe the water from a gushing fountain. He calls for frequent facial and chest sponging with ice water; demands rapid and vigorous fanning to the last. Streams and springs of former days are vividly portrayed in the thirsty patient's mind, and he is heard to cry out in most compassionate tones for only one glass from that sparkling stream. Water, water! is a frequent and pitiable cry. Champagne, water and all refreshing drinks on imbibition are instantaneously disgorged, as a rule. Retching is almost an ever present and distressing accompaniment.

Immense quantities of bile are not unfrequently ejected during emesis, and varies in color from an orange yellow to a spinach green, from a dirty golden seal to a black "coffee dregs." Frothy and viscid mucous is constantly disgorged. On the discharge of an enema, it will be found to contain muco-gelatinous particles characteristic to the "scrapings of hog intestines"; slight streaks of blood are occasionally to be observed, interlaced in the mucous. Tenacious and viscid mucous, similar in variety of colors to that of the vomit, is abundantly discharged, minus stercoraceous material on the administration of copious enema. Tympanitis is scarcely ever present, though not necessarily absent. The bowels are silent, but in favorable cases borborygm may be heard during or after the crisis.

Respiration is characteristically impeded, labored, intermittent, irregular, panting, and what I would like to term dicrotic, rapid, stridulous, and double panting, with a considerable pause, now and then. The heart sounds are sometimes tumultuous, loud, terribly confused and indistinct. In children its sounds resemble the rapid and irregular rubbing of the palms across each other.

The rapidity and irregularity of the pulse is so great at times, in adults as well as in children, that we are incapable to properly count it, probably ranging above 200. Now comes a nervous, haggard, restless expression, commingled with tranquility and comfort. The pulse now compressible, wiry, exceedingly intermittent, irregular, sometimes corded, dicrotic and finally lost, marbleized extremities, cold surface generally, profuse clammy sweats, vomiting ceasing, the mind clear, respiration intensely labored, and the patient departs this life generally propped up in bed. Duration, from six to seventy-two hours.

TREATMENT.—The virulence of malaria varies in intensity in the same locality during different years. The following tables will doubtless exhibit some interest. The first table is taken from families who live within a stone's throw of my own residence, and within the last four years:

| Family. | Cases. | Deaths. | Recoveries. |
|---|---|---|---|
| Redford............ | 5 | 2 | 3 |
| Ashe............... | 3 | 2 | 1 |
| Horlock ........ | 2 | 0 | 2 |
| Stewart ............ | 1 | 0 | 1 |
| Pender............. | 2 | 2 | 0 |
| Conoly............. | 3 | 0 | 3 |
| West .............. | 1 | 0 | 1 |
| Stickney ........... | 1 | 0 | 1 |
| Franklin........... | 1 | 0 | 1 |
| Total...... | 19 | 6 | 13 |

Visceral paresis occurred in each case.

Table of 1894, with diminished heroic treatment:

| Family. | Cases. | Deaths. | Recov. | Taken. | Died. |
|---|---|---|---|---|---|
| Pearson....... | 1 | 1 | 0 | July 2. | July 4. |
| Ackerman..... | 1 | 1 | 0 | July 14. | July 18. |
| Morgan (negro). | 1 | 1 | 0 | July 18. | July 19. |
| Maddox....... | 1 | 0 | 1 | July 29. | |
| Stoneham ..... | 1 | 1 | 0 | Aug. 3. | Aug. 3. |
| Reedy (negro).. | 1 | 1 | 0 | Aug. 7. | Aug. 7. |
| | — | — | — | | |
| | 6 | 5 | 1 | | |

The last case was taken at 12 m. and died at 2 p. m., perfectly conscious. I did nothing but administer quinine and whisky per rectum, eight or ten gr. mild. chlor. per oris, and ⅛ morphia hypdermically, as I could see no hopes for her. She was down town that a. m. and, as stated, taken at 12 m. The results of treatment will correspond to the ability of the physician to sustain cardiac action, peristalsis, and establish thorough cinchonism, therefore three vital principles devolve upon the practitioner: purgation, cinchonism and stimulation; the former will not infrequently be found impossible, until the paresis yields to the two latter principles, which sometimes will occur when rigidly pursued. It matters not the criticisms made concerning the administration of calomel, I will uphold, sustain, and laud its efficiency, as observation, experience and results develop a mortality reduction in its employment. The judicious administration of mild. chlor. is to be persisted in from the outset to and sometimes during convalescence. The drug meets the condition as near as the selection of any other single drug. The protiodide is utilized with very happy results also. A combination of the mild. chlor. protiodide, podophyl. sod. bicarb., with a few minims of ol. anise, or mint, made into powders, will, to some extent, allay nausea, and is quite effectual in arousing the secretions. A host of remedial agents could be readily suggested, but of doubtful utility. This may be followed by

Phos. sod................................ʒii

Sod. et pot. tart..........................ʒiii

Sol. cit. mag..........................q. s. Oj

To be taken at stated intervals. Nausea and vomiting is sometimes diminished by the use of ox. cerium in xx to xxx gr. doses. Creosote, carbol. acid and binn. sub. nit. may be beneficial. Morphia and atropine stands pre-eminent in controlling nausea, and especially the former, but must be cautiously administered on account of its contra-indications in regard to the secretions. Ice pellets are refreshing, ice cold water dashed in the face, and vigorous fanning, are very pleasant and beneficial in relief of nausea. The nit. strychnine hypodermically, in large and repeated doses, is paramount for three reasons: its antimalarial, muscular peristaltic, and stimulating influences. Sul. mag., introduced subcutaneously in the abdominal wall, I have found useful in assisting to unload the bowels. I have abandoned the idea of extensive blisters over the abdomen, as it is of doubtful utility in the extreme, and frequently does harm by influencing the kidney secretions. Sparteine is remarkably useful as a cardiac stimulant, and its dirurectic powers. Nitro-glycerine, for its rapid and powerful stimulating properties hypodermically, is to be greatly appreciated. Arsenic is quite applicable. Liquid peptonoids, also, for its palatable and nutritious therapeutic qualities. Referring to the salts of quinine, would say the bimur. is preferable. I use the three gr. tablets of S. & D., giving as many tablets, hypodermically, as may be desired at each insertion. My method is to employ the drug per oris, rectum, and hypodermically, simultaneously, if possible, and a repetition of the dose as may be indicated. The maximum amount I ever administered in twenty-four hours was ʒi without any evil results whatever. It appears to me that quinine antagonizes malaria, and vice versa, hence immense quantities may be taken with impunity. The same may be remarked of mercury. Unfortunately I have crippled patients for months by the extensive hypodermic use of bimur, though, if dissolved in 50 per cent. aa water and glycerine, the pain is greatly diminished on its introduction, and the improbability of abscesses. Whisky and chloroform, administered internally and hypodermically, are very beneficial in warding off the chill. Copious hot water enema, with fl. ext. aloe, are appropriate. Have used pure hot glycerine by the pint per rectum, with rectal tubes, without any effect, also hot ol. ricini in same quantities, with similar results. Ol. tiglii is ineffectual per oris. The most reliable enema consists in the introduction of a strong solution of sod. bicarb., followed by a solution of tartaric acid, immediately borborygm is heard, and probably resulting in fecal discharges. In 1894, I endeavored to depart from heroic treatment, and the result was a departure of most of my patients suffering with this disease.

