# Dr. Peeples’ Article

**Published:** 1895-07

**Authors:** 


IN DR. PEEPLES' article on Paralytic Miasmatic Fever in our last issue, it was stated that one dram of quinine is the maximum quantity of quinine ever given by him in twenty-four hours. The doctor writes us it should have been one ounce, instead of one dram. There were several minor typographical errors in the article, also, for which we tender apology.

